# Supplementary material for: Accurate genomic variant detection in single cells with primary template-directed amplification
Source: Proc Natl Acad Sci U S A. 2021 Jun 7;118(24):e2024176118. doi: 10.1073/pnas.2024176118 (PMC8214697; doi:10.1073/pnas.2024176118)
Supplement: Supplementary File [file pnas.2024176118.sapp.pdf]

PNAS
www.pnas.org

Supplementary Information for

Accurate Genomic Variant Detection in Single Cells with Primary Template-Directed Amplification

Veronica Gonzalez-Pena (Stanford University)
Sivaraman Natarajan (St. Jude Children's Research Hospital)
Yuntao Xia (Stanford University)
David Klein (Stanford University)
Rob Carter (St. Jude Children's Research Hospital)
Yakun Pang (Stanford University)
Bridget Shaner (St. Jude Children's Research Hospital)
Kavya Annu (St. Jude Children's Research Hospital)
Daniel K Putnam (St Jude Children's Research Hospital)
Wenan Chen (St. Jude Children's Research Hospital)
Jon Connelly (St. Jude Children's Research Hospital)
Shondra M Pruett-Miller (St. Jude Children's Research Hospital)
Xiang Chen (St. Jude Children's Research Hospital)
John Easton (St. Jude Children's Research Hospital)
Charles Gawad (Stanford University)

Charles Gawad
cgawad@stanford.edu

This PDF file includes:

Figures S1 to S16

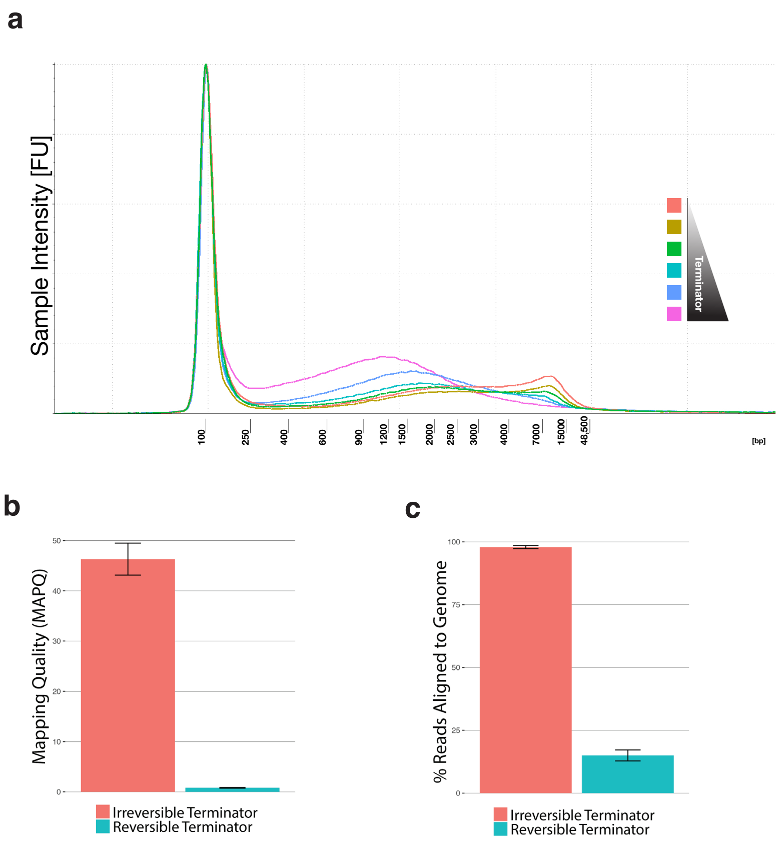

Fig. S1. The incorporation of irreversible terminators in PTA produces smaller, high-quality WGA products. a, Incorporation of alpha-thio-dideoxynucleotides terminate the extension step and decrease the size of the amplification products as seen in this terminator titration experiment showing amplicon size distribution on Tapestation 4200 high molecular weight tracings. (b and c), Irreversible terminators in PTA reactions produce high-quality amplification products with a 46.3+/-3.18 mapping quality, and 97.9+/-0.62% of reads mapped to the genome, while reversible terminators produce poor quality products with an overrepresentation of repetitive elements. (error bars represent one SD).

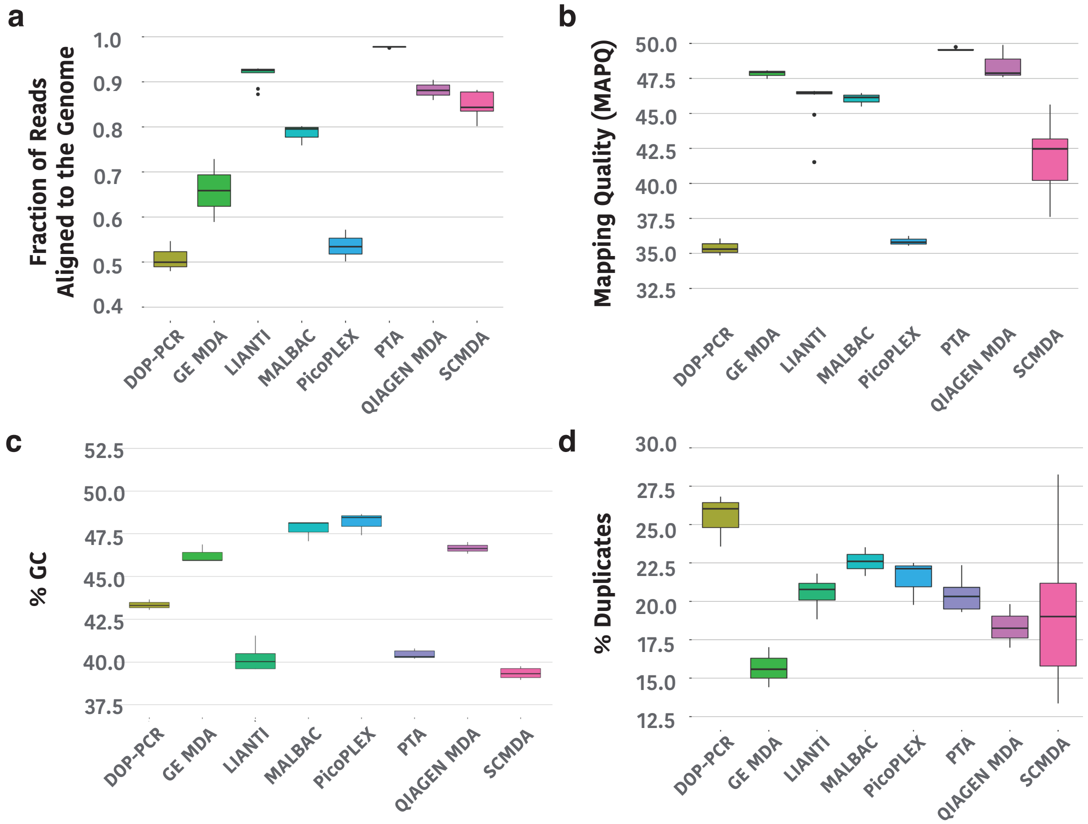

Fig. S2. Performance comparisons of PTA to the most common single-cell WGA methods. PTA and SCMDA were performed on 10 GM12878 cells each followed by library preparation and NGS. The resulting sequencing data was compared to data produced from single cells that had undergone amplification with DOP-PCR (n=3), GE MDA (n=3), Qiagen MDA (n=3), MALBAC (n=3), LIANTI (n=11), or PicoPlex (n=3) as part of the LIANTI study. a, Comparison of the fraction of reads aligned to the genome. b, Mapping quality. c, Percent GC content. d, PCR duplication rates. PTA data has the highest percent of reads aligned to the genome, the highest mapping quality, and lower GC content than most of the other methods. PCR duplication rates were similar across all methods. (for boxplots center line is the median; box limits represent upper and lower quartiles; whiskers represent 1.5x interquartile range; points show outliers).

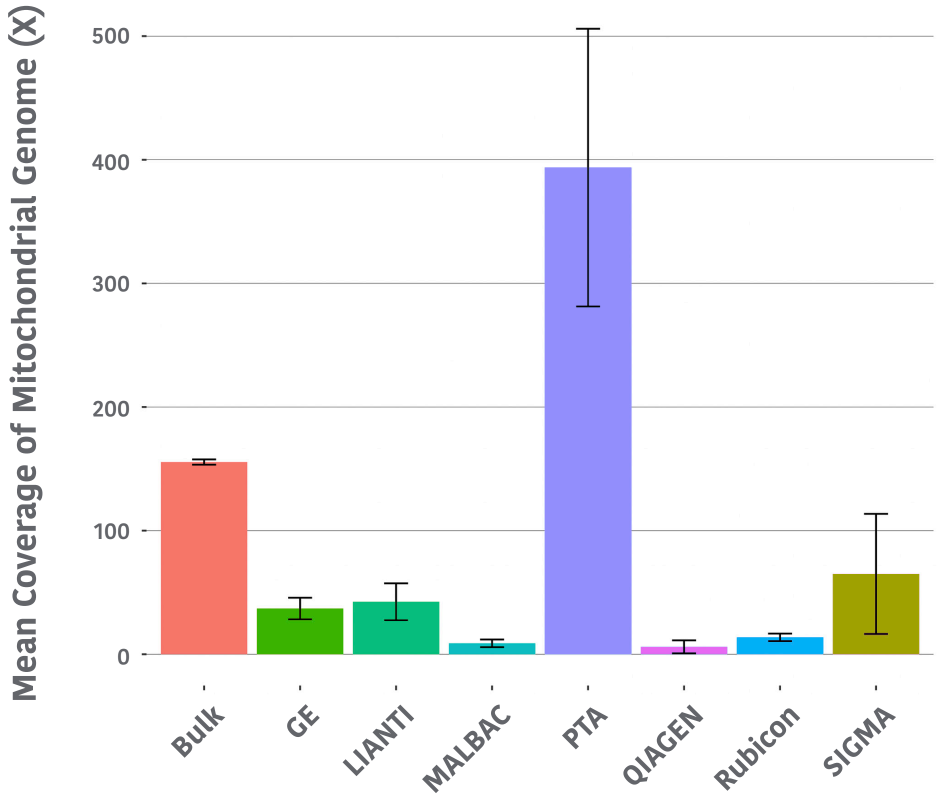

Fig. S3. Comparison of single cell mitochondrial genome coverage breadth with different WGA methods. All comparisons were done with the same number of sequencing reads; (error bars represent one SD).

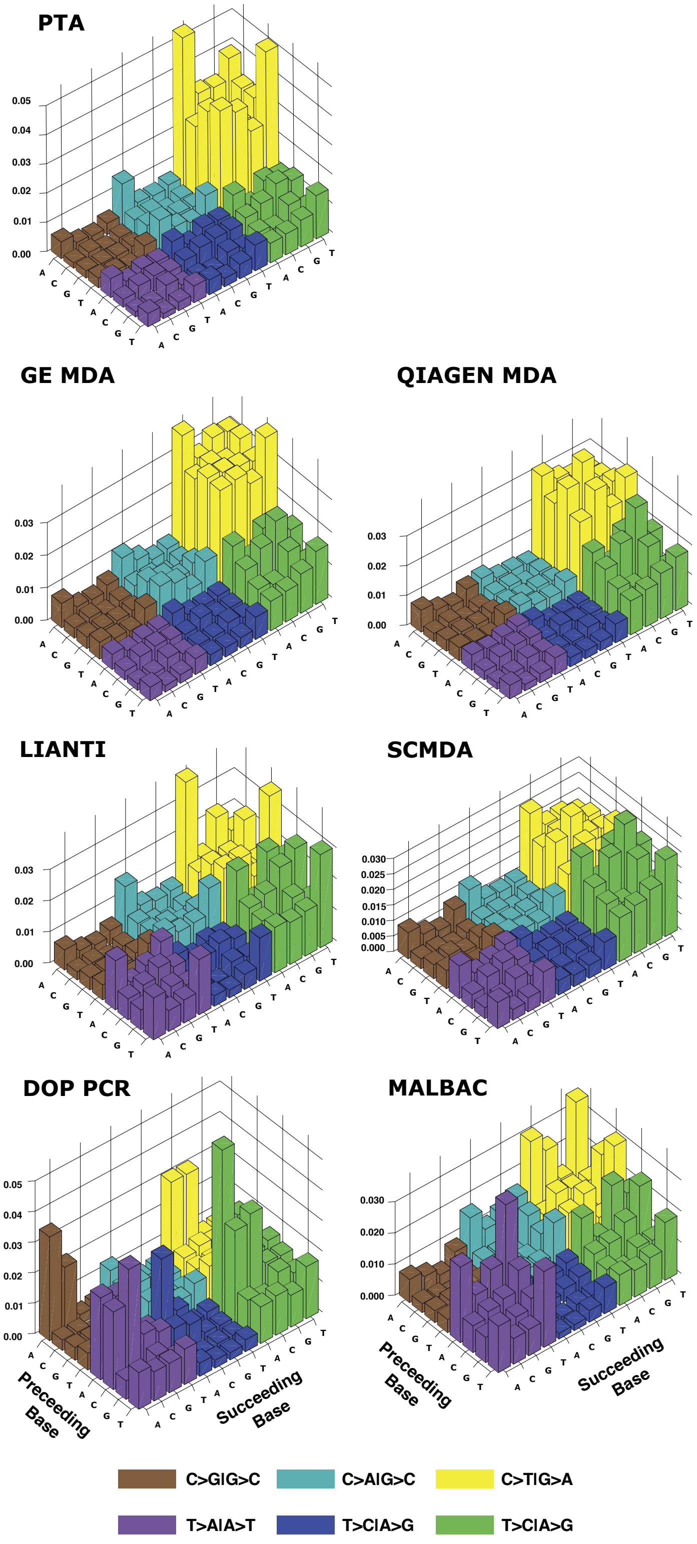

Fig. S4. Trinucleotide base change patterns in false positive SNVs. Base change patterns seen in false positive calls appear to be polymerase-dependent with methods using an isothermal polymerase showing a preference for C to T (G to A) changes.

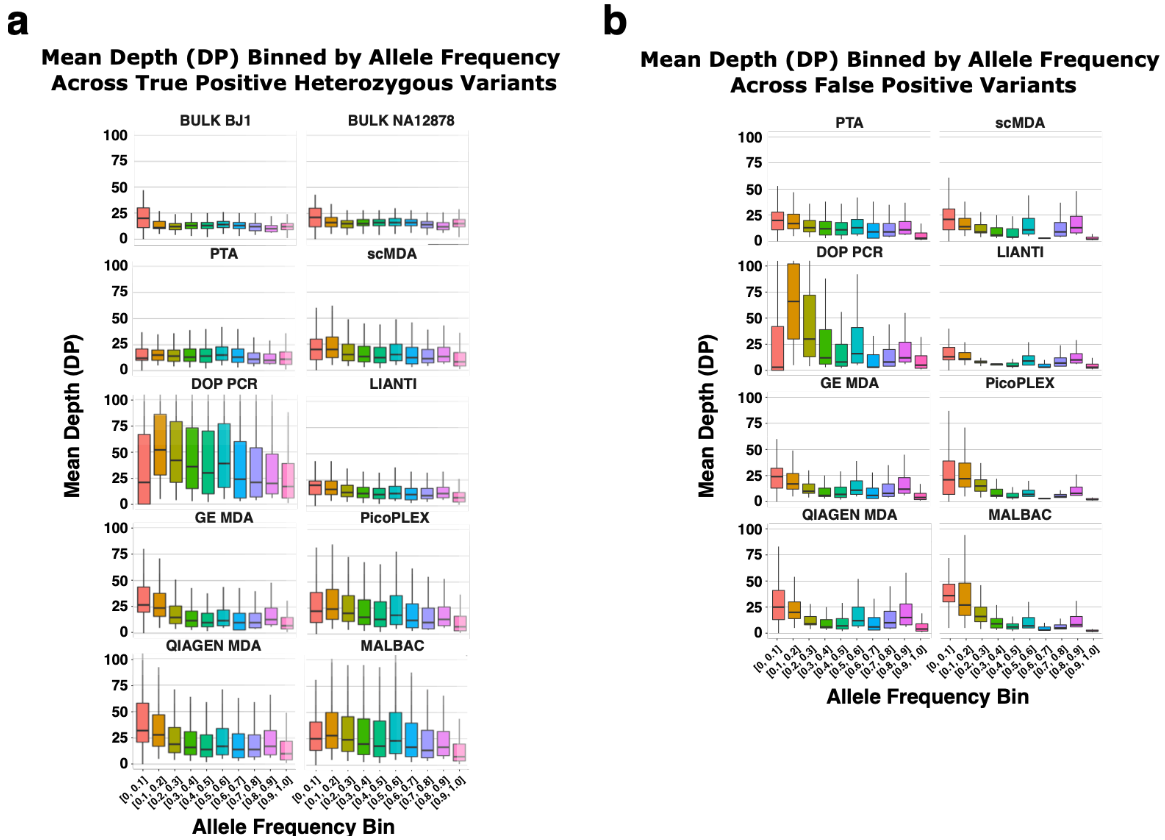

Fig. S5. Coverage depth at increasing variant allele frequency bin for true positive (a) and false positive (b) variant calls for each of the WGA methods.

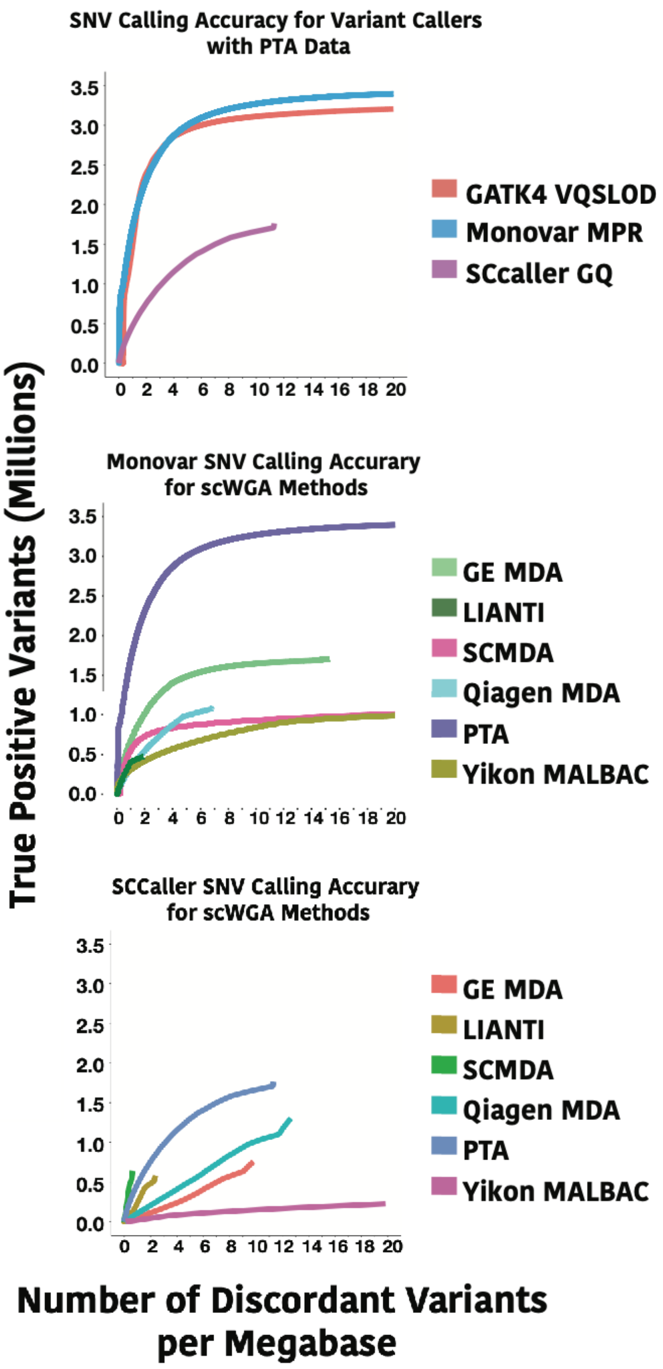

Fig. S6. Comparison of SNV calling accuracy for different single cell variant callers.

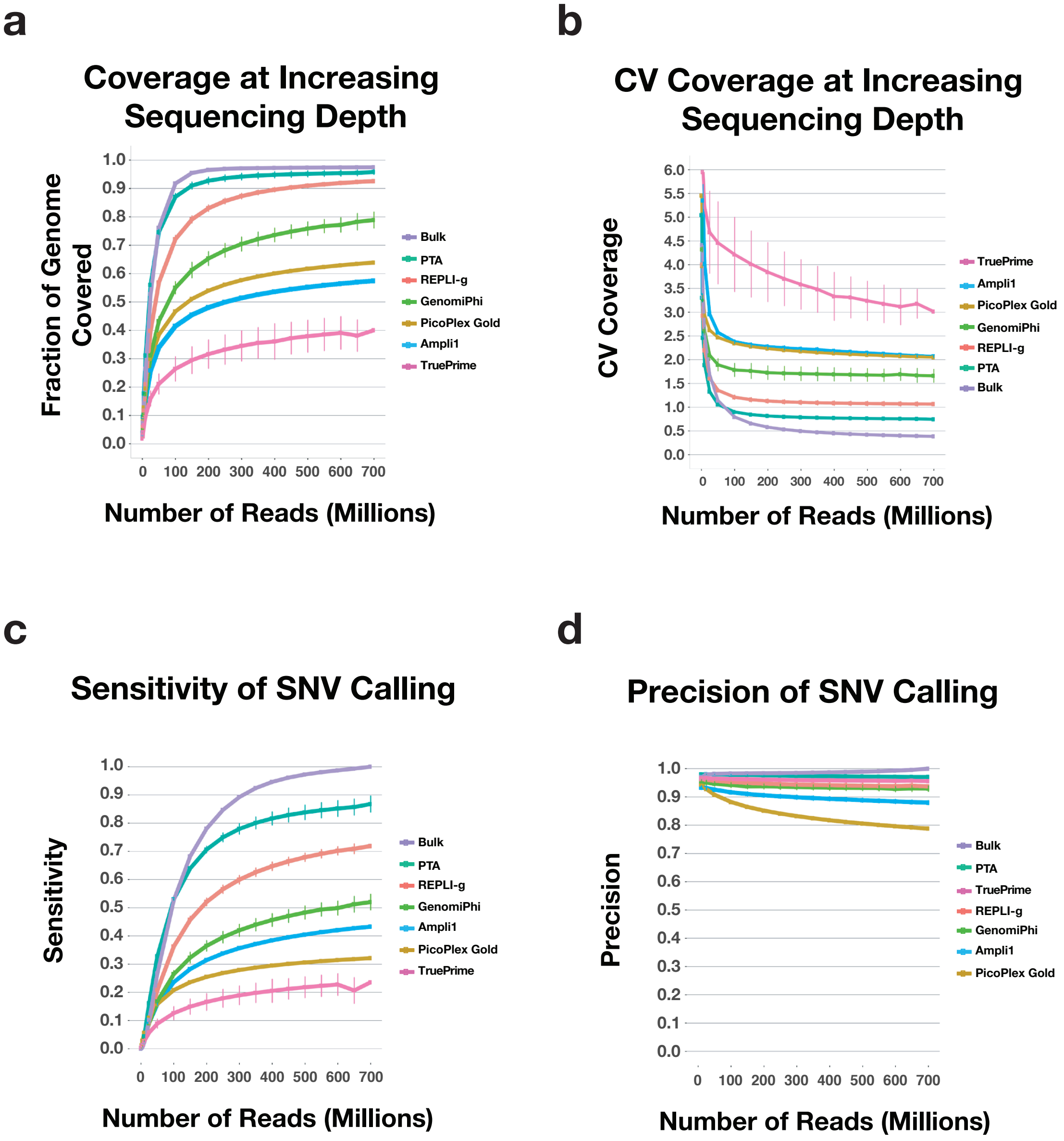

Fig. S7. Alignment and SNV calling metrics in primary leukemia cells at increasing sequencing depth using low temperature lysis PTA a, Coverage breadth b, CV coverage c, SNV calling sensitivity d, SNV calling precision (n=5 for each method, error bars represent one SD).

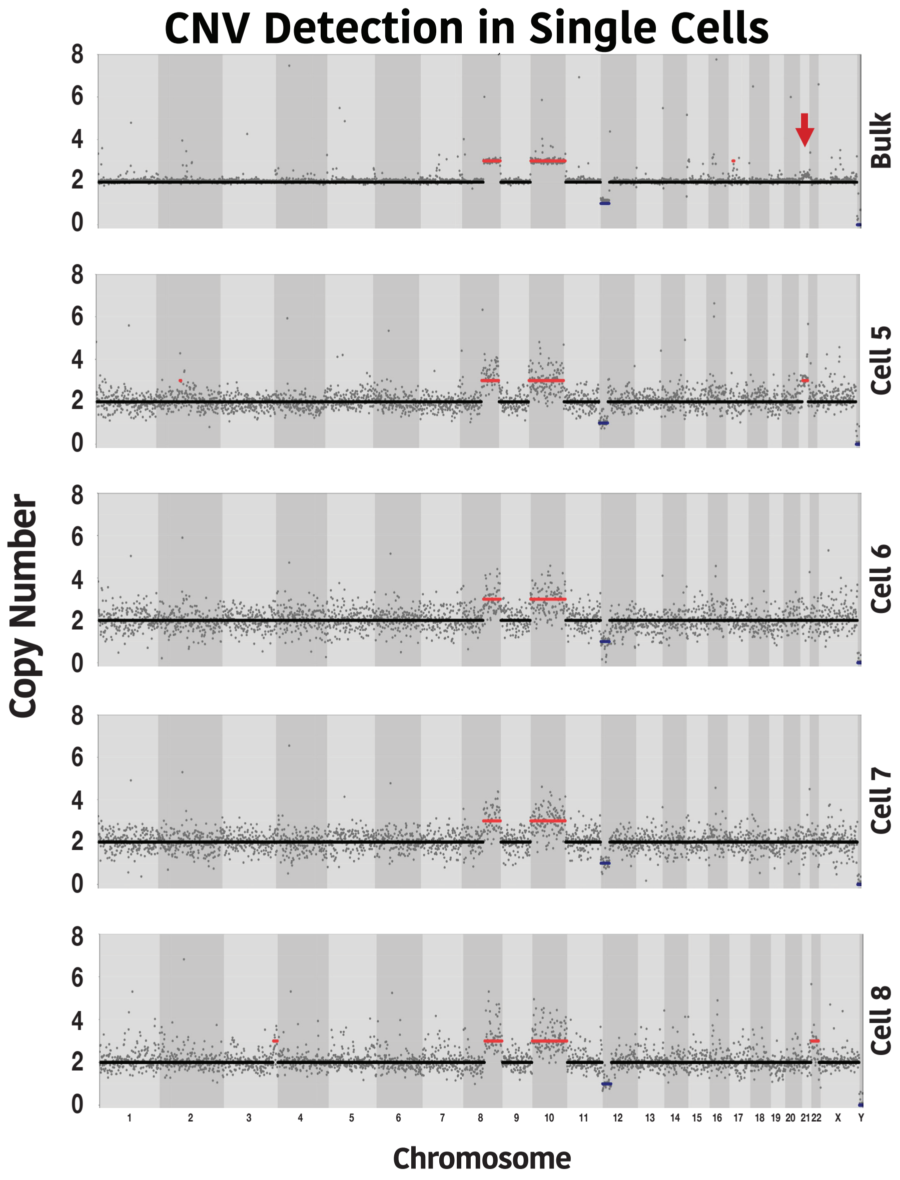

Fig. S8. Additional single cell CNV profiles for cells presented in Fig. 3h.

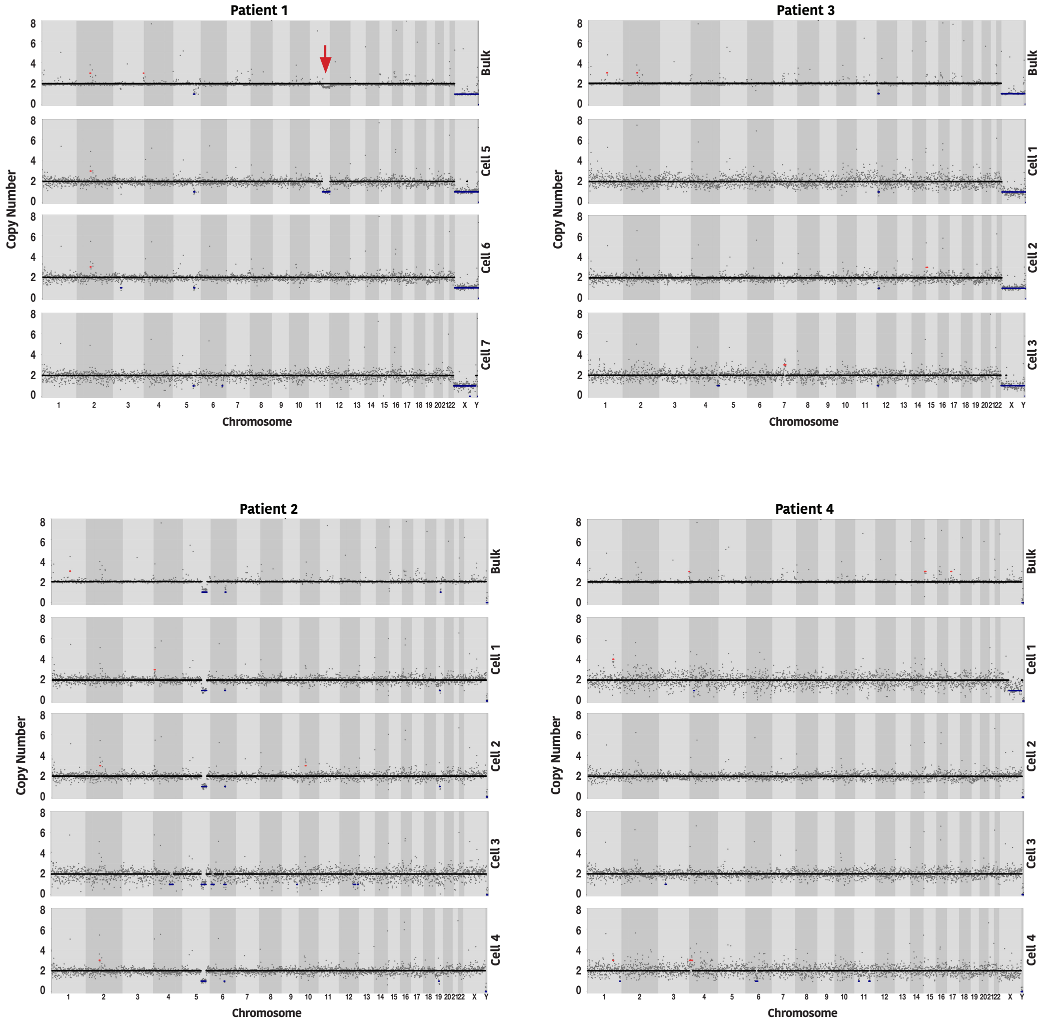

Fig. S9. Bulk and single cell CNV profiles for four additional patient samples.

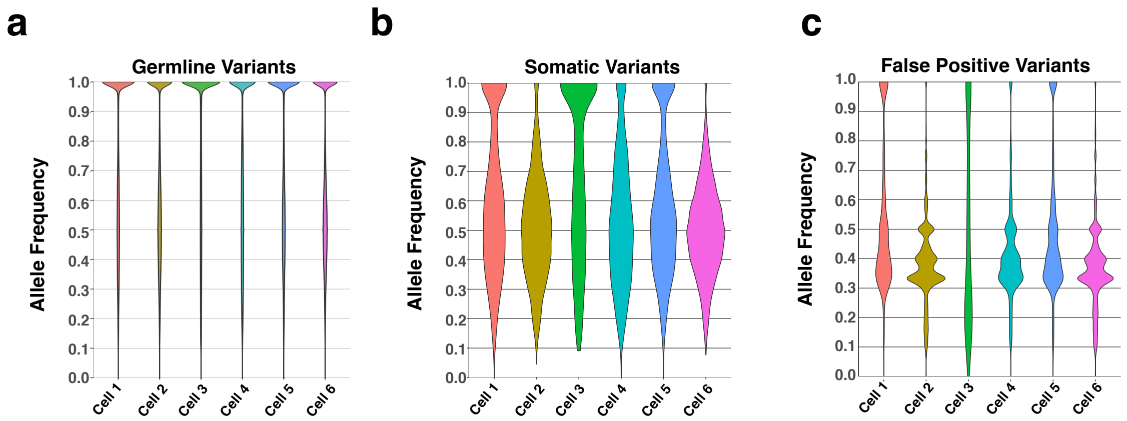

Fig. S10. Allele frequency distributions using standard filtering for a, Germline variants, b, Somatic variants, and c, False positive variants.

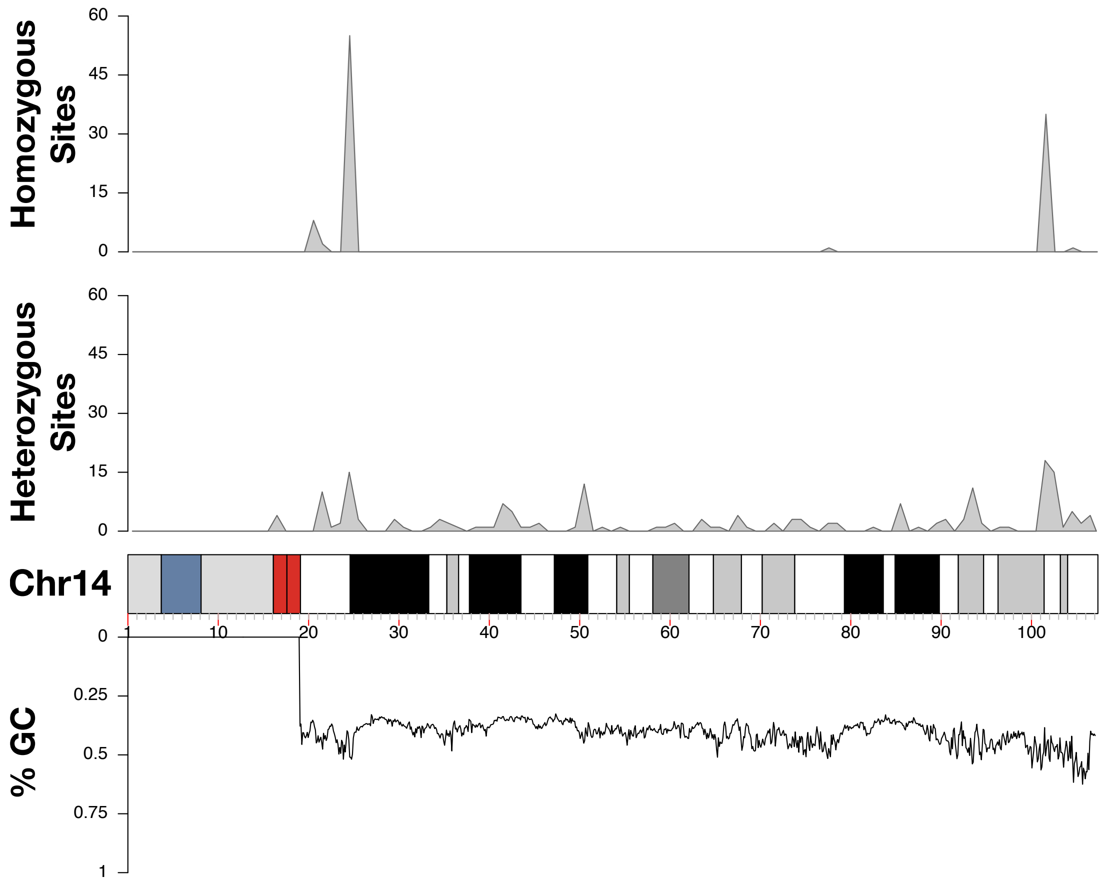

Fig. S11. Density of homozygous or heterozygous false positive variant calls across chromosome 14 (which had the largest number of false positive calls). Mean GC content at 100 Kb intervals runs below the karyogra

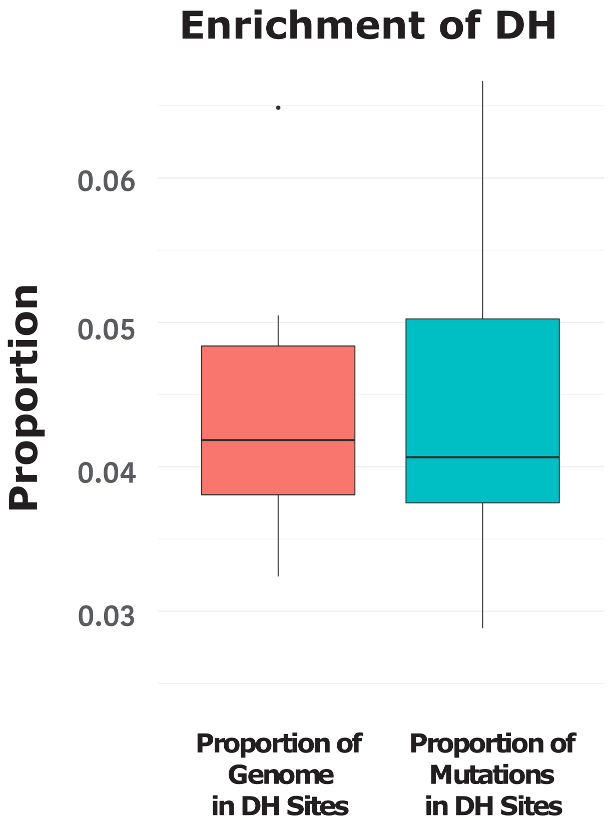

Fig. S12. Proportion of ENU induced mutations in DNase I hypersensitive (DH) sites. DH sites in CD34+ cells previously catalogued by the Roadmap Epigenomics Project were used to investigate whether ENU mutations are more prevalent in DH sites which represent sites of open chromatin. No significant enrichment in variant locations at DH sites was identified. Further, no enrichment of variants restricted to cytosines was observed in DH sites. (for boxplots center line is the median; box limits represent upper and lower quartiles; whiskers represent 1.5x interquartile range; points show outliers).

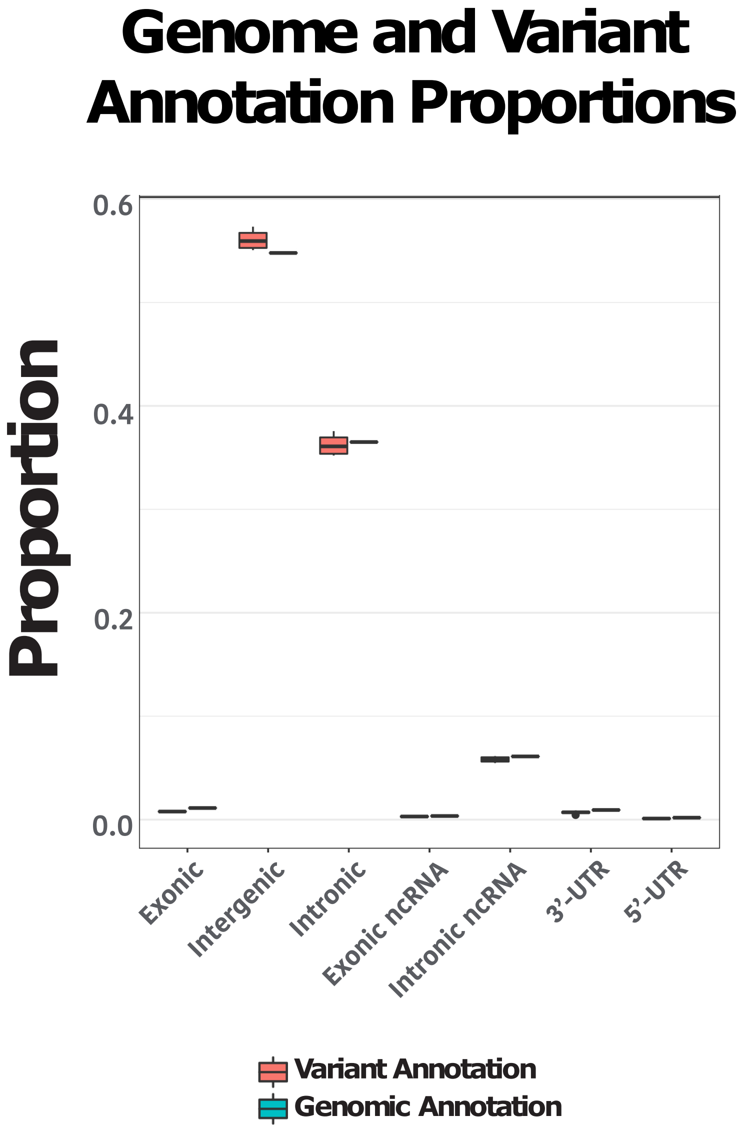

Fig. S13. Proportion of ENU-induced mutations in genomic locations with specific annotations. No specific enrichment was seen in specific annotations for variants in each cell relative to the proportion of the genome each annotation comprises. (for boxplots center line is the median; box limits represent upper and lower quartiles; whiskers represent 1.5x interquartile range; points show outliers).

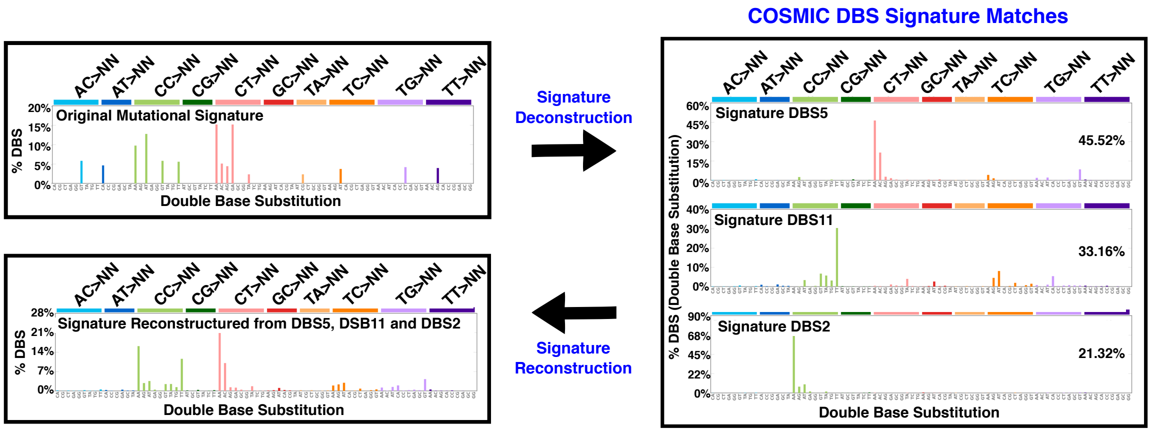

Fig. S14. Double base substitution signature for ENU-induced variants in CD34+ cord blood cells.

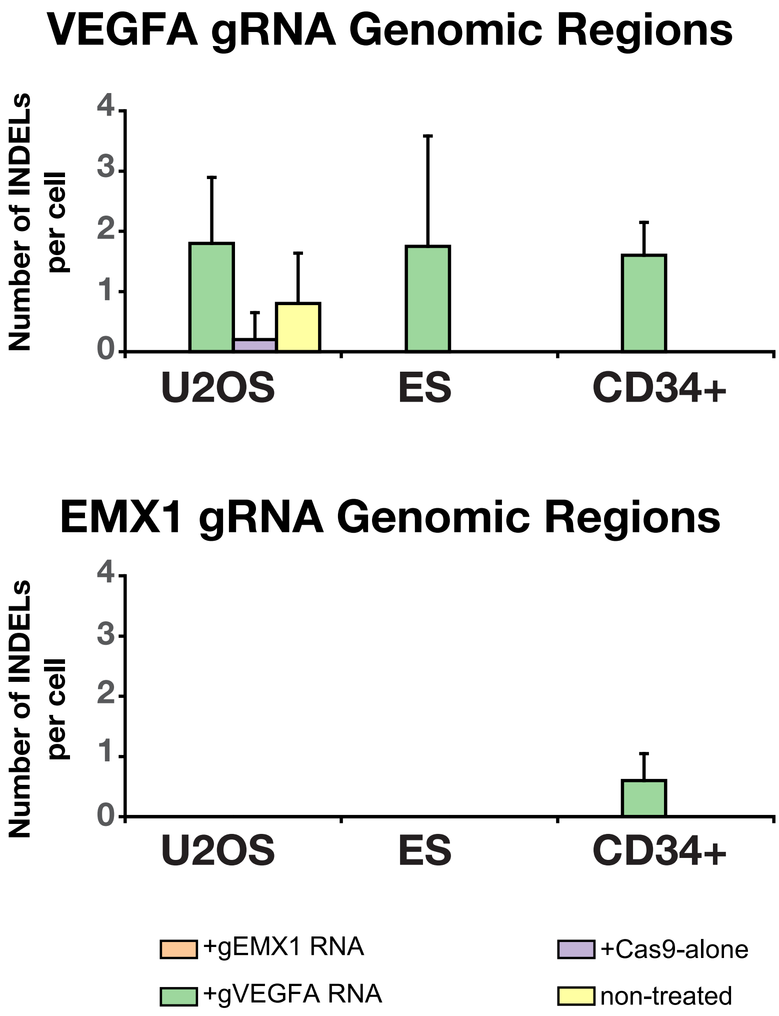

Fig. S15. Removing non-recurrent single base pair insertions improves the precision of off-target detection. Each control or experimental cell type underwent indel calling requiring no more than five mismatches to either the VEGFA or EMX1 guide RNA sequence. Off-target events specifies which genomic region the gRNA had to match while the gRNA or control listed in the key specify which gRNA that cell received. Instances where the indel is called in a genomic region that does not match the gRNA received by that cell are presumed to be false positives.

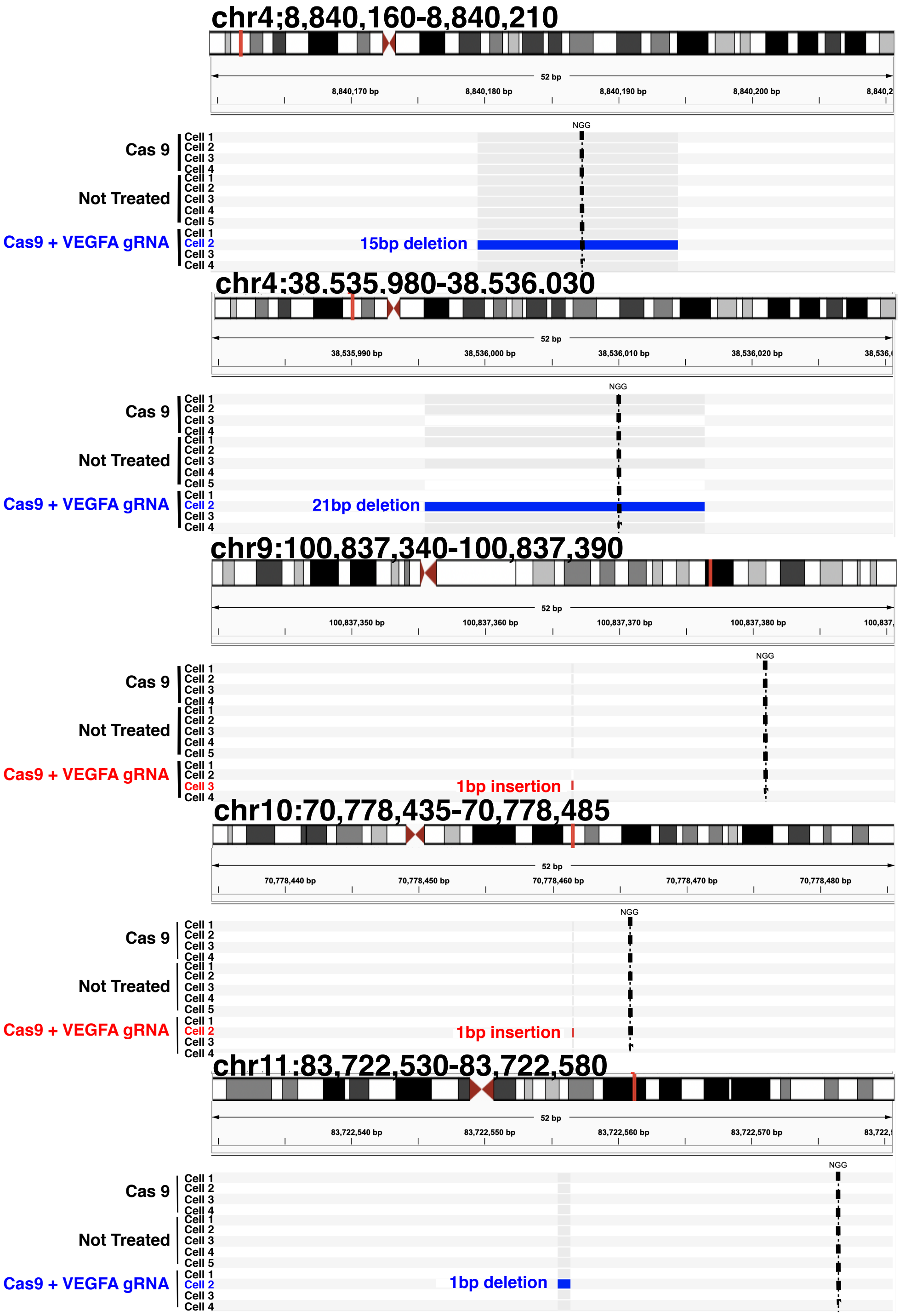

Fig. S16. Confirmation of five VEGFA CRISPR off-target sites in ES cells using targeted resequencing.
